# *Aspergillus Sydowii* Marine Fungal Bloom in Australian Coastal Waters, Its Metabolites and Potential Impact on *Symbiodinium* Dinoflagellates

**DOI:** 10.3390/md14030059

**Published:** 2016-03-16

**Authors:** Aiko Hayashi, Andrew Crombie, Ernest Lacey, Anthony J. Richardson, Daniel Vuong, Andrew M. Piggott, Gustaaf Hallegraeff

**Affiliations:** 1 Institute for Marine & Antarctic Studies, University of Tasmania, Hobart, Tasmania 7004, Australia; aiko.hayashi@utas.edu.au; 2 Microbial Screening Technologies, Building A, 28-54 Percival Rd, Smithfield NSW 2164, Australia; acrombie@microbialscreening.com (A.C.); elacey@microbialscreening.com (E.L.); dvuong@microbialscreening.com (D.V.); 3 CSIRO Marine & Atmospheric Research, Ecosciences Precinct, Brisbane, Queensland 4102, Australia; anthony.richardson@csiro.au; 4 Centre for Applications in Natural Resource Mathematics, School of Mathematics and Physics, University of Queensland, St Lucia, Queensland 4072, Australia; 5 Department of Chemistry and Biomolecular Sciences, Macquarie University, NSW 2109, Australia; andrew.piggott@mq.edu.au

**Keywords:** *Aspergillus sydowii*, *Symbiodinium*, secondary metabolites, sydonic acid, sydowinin, sydowinol, sydonol, coral disease, maximum quantum yield (*F_v_*/*F_m_*)

## Abstract

Dust has been widely recognised as an important source of nutrients in the marine environment and as a vector for transporting pathogenic microorganisms. Disturbingly, in the wake of a dust storm event along the eastern Australian coast line in 2009, the Continuous Plankton Recorder collected masses of fungal spores and mycelia (~150,000 spores/m^3^) forming a floating raft that covered a coastal area equivalent to 25 times the surface of England. Cultured *A. sydowii* strains exhibited varying metabolite profiles, but all produced sydonic acid, a chemotaxonomic marker for *A. sydowii*. The Australian marine fungal strains share major metabolites and display comparable metabolic diversity to Australian terrestrial strains and to strains pathogenic to Caribbean coral. Secondary colonisation of the rafts by other fungi, including strains of *Cladosporium*, *Penicillium* and other *Aspergillus* species with distinct secondary metabolite profiles, was also encountered. Our bioassays revealed that the dust-derived marine fungal extracts and known *A. sydowii* metabolites such as sydowic acid, sydowinol and sydowinin A adversely affect photophysiological performance (*F_v_*/*F_m_*) of the coral reef dinoflagellate endosymbiont *Symbiodinium*. Different *Symbiodinium* clades exhibited varying sensitivities, mimicking sensitivity to coral bleaching phenomena. The detection of such large amounts of *A. sydowii* following this dust storm event has potential implications for the health of coral environments such as the Great Barrier Reef.

## 1. Introduction

In a previous publication [1], we reported an extensive *Aspergillus sydowii* marine fungal bloom in the wake of an Australian dust storm event in 2009. The Continuous Plankton Recorder (CPR) collected masses of fungal spores and mycelia, estimated to be up to 150,000 spores per m^3^, between Brisbane and Sydney, an area equivalent to 25 times the surface of England. Fungal spores and mycelia were identified as *Aspergillus sydowii* using molecular sequencing of three different genes (large-subunit rRNA gene, internal transcribed spacer and beta tubulin) with 99%–100% match. *A. sydowii* has been widely claimed to cause aspergillosis in Caribbean gorgonian corals.

*Aspergillus* species are pathogenic to a wide range of organisms [2]. In marine environments, *A. sydowii* is characterised as a causative agent of aspergillosis of sea fan corals, based on morphological, physiological and nucleotide sequence analysis, and Koch’s postulate [3,4]. *A. sydowii* is essentially a terrestrial organism, unable to sporulate and complete its life cycle in seawater [3]. Symptoms of aspergillosis include small lesions of necrotic tissue with purple halos [3], resembling the pathology of coral bleaching and hence suggesting an impact on *Symbiodinium* dinoflagellate symbionts. This fungal species is known to infect several species of octocorals [5], and has caused 20%–90% sea fan mortality in the Florida Keys [6]. Due to the significant mortality and subsequent changes in the coral community structure, research focus needs to shift from the etiology of the disease to greater understanding of the interactions among causal pathogen, coral and their endosymbionts *Symbiodinium* [7].

A putative virulence factor was initially proposed for *A. sydowii* strains isolated from diseased sea fan corals, although molecular genetic analysis reveals no clear differences between pathogenic and non-pathogenic strains [8,9]. Furthermore, there were no clear differences in temperature tolerance, susceptibility of coral host crude extract and carbon source utilization patterns [10]. However, Geiser *et al.* [8] found that when sea fans were inoculated with virulent isolates from affected sea fans, all showed typical symptoms of aspergillosis at the point of inoculation, whereas sea fans with avirulent isolates showed no symptoms.

The role of secondary metabolites in pathogenesis has been largely unexplored [5]. While over thirty metabolites from *A. sydowii* have been reported in the literature, most exhibit chemistry related to sydonic acid and sydowinin [11,12,13,14,15]. Comparative HPLC analysis of marine pathogenic and non-pathogenic *A. sydowii* strains demonstrate overlapping metabolite profiles, but none were attributable to specific *A. sydowii* metabolites [16]. Therefore, the composition of the metabolite profiles and their relationship to pathogenesis remain unclear.

The aim of this study was to examine metabolic profiles of new fungal isolates from the 2009 Australian dust storm plankton silks and compare their metabolic profiles with those from other sources including terrestrial habitats and diseased Caribbean sea fan corals. We also re-evaluate the fungal diversity in the 2009 plankton rafts, and assess the impacts of *A. sydowii* metabolites on various strains of the dinoflagellate coral endosymbiont, *Symbiodinium*.

## 2. Results

### 2.1 Australian Terrestrial A. Sydowii Strains

**Figure 1:**
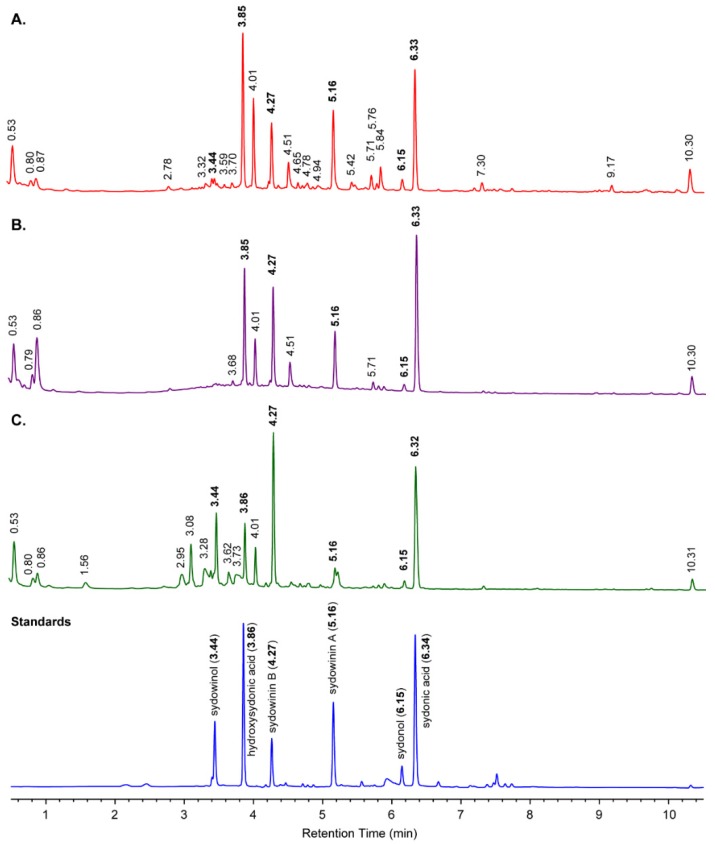
Comparison of HPLC traces (210 nm) of secondary metabolites of methanolic extracts from selected strains of *A. sydowii* grown on rice: (**A**) Terrestrial strain FRR5068; (**B**) Marine strain ASBS; (**C**) US Marine pathogenic strain FK1; **Standards**: metabolites isolated from *A. sydowii* and maintained in MST’s metabolite library. The HPLC trace has been truncated from 0.5 to 10.5 min to remove polar endogenous and primary metabolites (<0.5 min) in the solvent front and non-polar grain oils (>10.5 min.).

**Figure 2:**
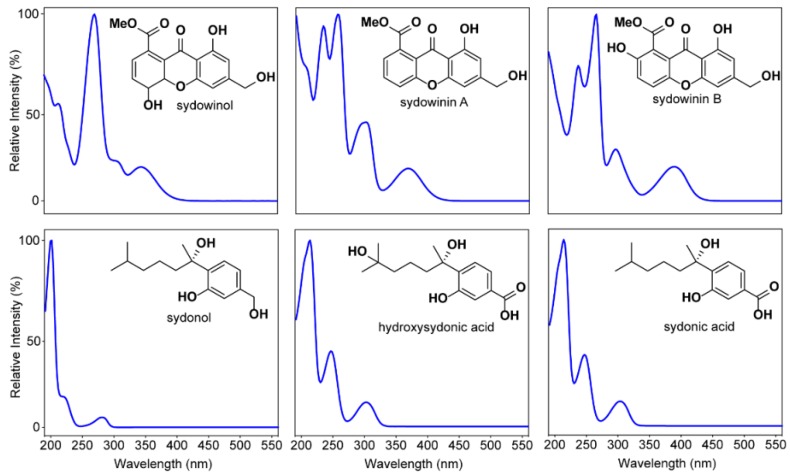
The structures and UV-Vis spectra of *A. sydowii* metabolite standards.

The metabolite consensus for *A. sydowii* was developed by examining seven strains held in the Food Research North Ryde (FRR) Fungal Culture Collection. Five of the strains were sourced from Australian terrestrial locations and two from Indonesian terrestrial locations (Supplementary Material, Table S1). Cultivation of the strains in liquid and on agar gave low levels of secondary metabolites, with yeast extract and sucrose (YES) agar consistently superior in promoting abundance and diversity, consistent with previous reports [16]. Further increases in production (5- to 10-fold) were achieved by cultivation on grains, in particular, rice. Strains showed some variation, but overall gave a consistent profile of major metabolites (Supplementary Material, Figure S1). Strain FRR5068 was considered representative of *A. sydowii* and the co-metabolite profile of this species *in sensu*. Metabolites from *A. sydowii* (FRR5068) grown on rice for 14 days and extracted with methanol were separated by gradient HPLC using diode array detection (DAD) and mass spectrometry (MS) (Figure 1A). Assessment of the co-metabolite diversity was undertaken using UV detection at 210 nm, with 78 discrete secondary metabolites being responsible for 99.5% of the total area under metabolite peaks (AUC) from 0.5 to 10.5 min. Analysis of the percent abundance of the metabolites revealed a hyper-dispersed distribution, with most metabolites present in trace amounts (Supplementary Material, Table S2) and only 25 metabolites responsible for 90% of total metabolite AUC. The metabolite distribution can be described in terms of the metabolite’s polarity on reverse phase (C_18_) HPLC. Four unidentified polar metabolites eluting 0.5–1.0 min accounted for 8.9% of the total metabolite AUC. These compounds exhibited simple UV-Vis spectra (λ_max_ 200–210 nm), but did not ionize in the mass spectrometer, precluding identification. The intermediate-eluting peaks from three to seven minutes contained a complex series of metabolites dominated by variants of three distinct UV-Vis spectral classes (Figure 2). Comparison of the retention times, UV-Vis spectra and MS data of a mixture of known *A. sydowii* metabolites (Figure 1, Standards) identified five of the six major metabolites as sydowinol (*t*_R_ 3.44 min, 0.51%; λ_max_ 212, 268, 301, 343 nm; ESIMS *m*/*z* [M − H]^−^ 317, [M + H]^+^ 319), hydroxysydonic acid (*t*_R_ 3.85 min, 16.3%; λ_max_ 213, 248, 303 nm; ESIMS *m*/*z* [M − H]^−^ 281, [M + Na]^+^ 305), sydowinin B (*t*_R_ 4.27 min, 6.9%; λ_max_ 237, 265, 297, 390 nm; ESIMS *m*/*z* [M − H]^−^ 315, [M + H]^+^ 317), sydowinin A (*t*_R_ 5.16 min, 10.8%; λ_max_ 206, 234, 258, 300, 370 nm; ESIMS *m*/*z* [M − H]^−^ 299, [M + H]^+^ 301), sydonol (*t*_R_ 6.15 min, 1.6%; λ_max_ 202, 219, 280 nm; ESIMS *m*/*z* [M − H]^−^ 251) and sydonic acid (*t*_R_ 6.33 min, 16.0%; λ_max_ 215, 248, 304 nm; ESIMS *m*/*z* [M − H]^−^ 265, [M + Na]^+^ 289). The sixth metabolite, eluting at 4.01 min and constituting 9.1% of the metabolite AUC, exhibited a sydonic acid UV-Vis spectrum and molecular weight 282 amu, and is tentatively considered an isomer of hydroxysydonic acid. The known metabolites constitute 50.4% of the total metabolite AUC. The remaining eight major metabolites could be identified by their respective UV-Vis spectra as analogues of sydonic acid, sydowinin or sydonol, while six metabolites could not be assigned to a specific chemical based on their UV-Vis spectra.

The non-polar region from 7 to 10.5 min contained two metabolites, a sydowinin analogue (7.30 min, 1.22%) and a sydonol analogue (9.17 min, 0.88%), with the final metabolite, a fatty acid (λ_max_ < 200 nm) hydrolysed from grain oils (10.30 min, 4.04%).

### 2.2. Australian Marine A. Sydowii ASBS Strain

The *A. sydowii* ASBS strain was isolated by towing a Continuous Plankton Recorder (CPR) instrument through a microbial raft located at 28.424°S, 153.811°E off the Australian mainland after the 2009 dust storm [1]. Genetic sequences of spores from the CPR silks indicated the strain to be a 99%–100% match to *A. sydowii* [1]. The metabolite profile of *A. sydowii* ASBS grown on rice for 14 days and extracted with methanol is presented in Figure 1B. Visual inspection of Figure 1A,B show a strong overlap in the both the polar and intermediate polar regions of the HPLC traces. Assessment of the co-metabolite diversity revealed 63 metabolites were responsible for 99.5% of the total metabolite AUC of the HPLC trace from 0.5 to 10.5 min. Like FRR5068, the metabolite distribution was hyper-dispersed, with most metabolites present in trace amounts and only 13 metabolites responsible for 90% of the total metabolite AUC (Supplementary Material, Table S3). More than half (57.3%) of the co-metabolite distribution comprised five of the known *A. sydowii* metabolites: hydroxysydonic acid (3.85 min, 13.8%), sydowinin B (4.27 min, 11.6%), sydowinin A (5.16 min, 8.0%), sydonol (6.15 min, 1.1%) and sydonic acid (6.32 min, 24.0%). Retention times and UV-Vis spectra of each metabolite were consistent with the authentic standards.

### 2.3. US Marine A. Sydowii FK1 Strain

The *A. sydowii* FK1 strain was identified as the causal pathogen of *Gorgonia ventalina* from Key West, Florida, USA. The metabolite profile of *A. sydowii* FK1 grown on rice for 14 days and extracted with methanol exhibited less metabolic diversity than the Australian *A. sydowii* cultures (Figure 1C), with only 48 metabolites being responsible for 99.5% of the total metabolite AUC and 17 metabolites responsible for 90% of the AUC (Supplementary Material, Table S4). The HPLC was nonetheless dominated by the known metabolites with sydowinol (3.44 min, 8.5%), hydroxysydonic acid (3.86 min, 6.1%), sydowinin B (4.27 min, 18.6%), sydowinin A (5.16 min, 1.1%), sydonol (6.15 min, 0.92%) and sydonic acid (6.32 min, 20.8%) constituting 55% of the metabolite AUC. The relative abundance of the known metabolites is different from the Australian strains, with increased levels of sydowinol and sydowinin B at the expense of hydroxysydonic acid and sydowinin A. The strain also displays high abundance of unidentified metabolites not observed in the Australian strains, eluting from 2.95 to 3.73 min. The six metabolites constitute nearly 17% of the co-metabolite profile and their respective retention times (λ_max_ and *m*/*z*) are a chlorinated acidic metabolite eluting at 2.95 min (λ_max_ 198, 210 s, 268, 300 s nm, ESIMS *m/z* [M − H]^−^ 377/379) together with a dichloro analogue eluting at 3.62 min (λ_max_ 198, 220 s, 270, 330 s nm, ESIMS *m*/*z* [M − H]^−^ 411/413), three polar neutral metabolites eluting at 3.08 min (λ_max_ 198, 220, 265 nm, ESIMS *m/z* [M − H]^−^ 230, [M + H]^+^ 232), 3.28 min (λ_max_ 212, 226 s, 242 s, 298 s, 313 s, 331 nm, ESIMS *m/z* [M − H]^−^ 235, [M + H]^+^ 237) and 3.36 min (λ_max_ 192, 225 s, 240, 258 s, 328 nm, ESIMS *m/z* [M − H]^−^ 235, [M + H]^+^ 237). A final metabolite eluting as a broad peak at 3.73 min failed to provide useable MS ionisation, but possessed a highly characteristic UV spectrum (λ_max_ 230, 254, 278 s, 333 nm). The metabolites appear unique among *A. sydowii* strains. Further, the λ_max_ values are inconsistent with those for the *A. sydowii* co-metabolites described in the literature.

### 2.4. Fungal Strains from the 2009 CPR Silks, and Metabolites in CPR Silk Materials

**Figure 3:**
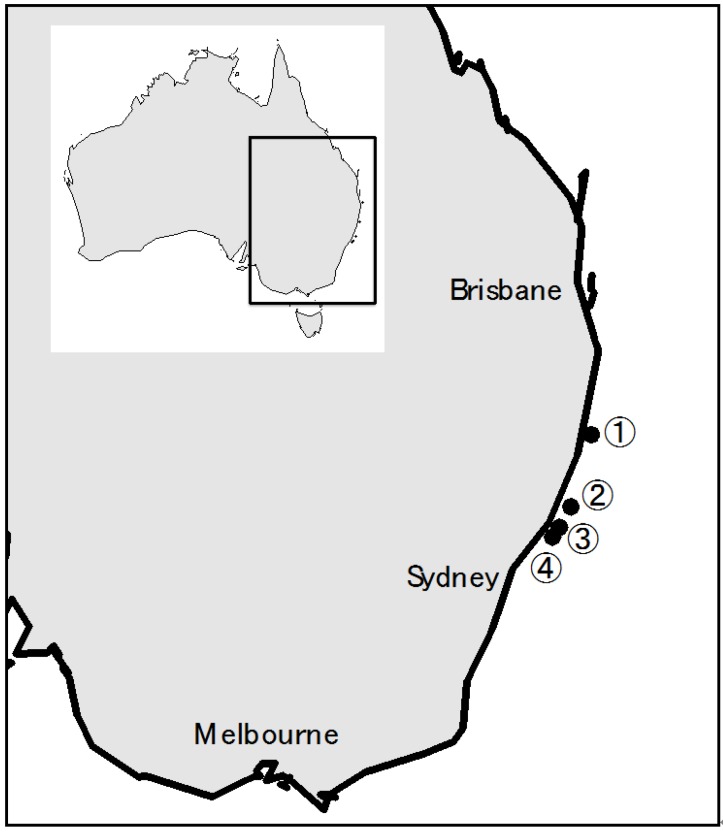
The distribution of fungal species in the 2009 dust storm. Latitude and longitude of each location (circled number from 1 to 4) are following: 30.617–30.703°S, 153.425–153.401°E (location 1), 32.238–32.319°S, 152.884–152.842°E (location 2), 32.701°S, 152.579°E (location 3), 32.917–32.989°S, 152.393–152.331°E (location 4).

Further investigation of the 2009 CPR silks supported the dominance of *A. sydowii*, but also detected a few minor species: 73.7% *A. sydowii*; 10.5% unknown species (based on HPLC analysis); 7.9% *Aspergillus* sp.; 5.3% *Penicillium* sp. (Table 1 and Figure 3). These strains were isolated using half strength malt extract agar (MEA) and tetracycline, and included *Cladosporium* sp. (from location 4) and *Penicillium* sp. (from location 3).

The isolated *Penicillium* strains produced both known metabolites, such as rugulosin and previously unencountered co-metabolite profiles, the latter suggesting a novel species. Additional unidentified species producing unknown metabolites were also encountered from locations 1 and 4. Further genetic and metabolic characterisation of those unknown species is in progress. Additionally, *Aspergillus* sp. producing sterigmatocystin was detected. HPLC analyses of isolated *A. sydowii* indicated varying metabolite profiles and some produced unknown metabolites on MEA; however, all isolates produced sydonic acid. Their metabolite profiles exhibited some degree of overlap with some, but not all, terrestrial *A. sydowii* strains.

Direct extraction of CPR silk materials provided a different perspective on the dominance of *A. sydowii*. The chromatographs of silks from 32.238–32.319°S, 152.884–152.842°E (location 2) and 32.701°S, 152.579°E (location 3) exclusively detected *Pseudomonas* phenazine metabolites, tubermycin (λ_max_ 248, 344 s, 370 nm) and oxychlorophine (λ_max_ 248, 344 s, 370 nm), while *A. sydowii* metabolites were present in only trace amounts (Supplementary Material Figure S2).

### 2.5. Effect of Terrestrial and Marine A. Sydowii Crude Extracts on *Symbiodinium* Photo-Physiological State

**Figure 4:**
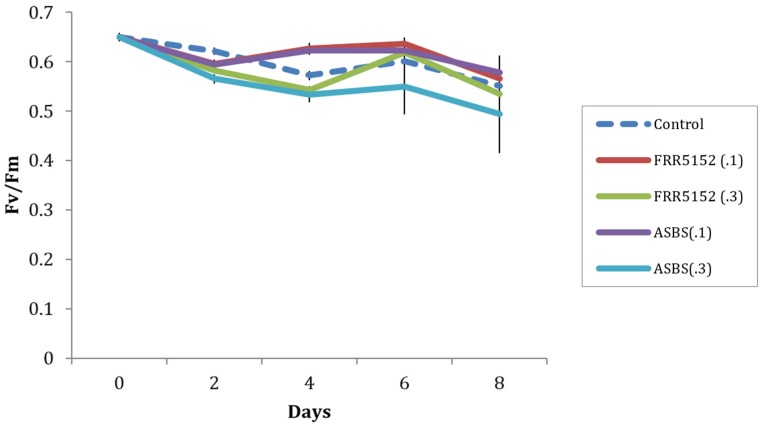
Effect of terrestrial (FRR5152) and 2009 dust originated *A. sydowii* (ASBS) crude extracts on CS156 *Symbiodinium* maximum fluorescent yield (*F_v_*/*F_m_*) during an 8-day period. Error bars represent sample standard deviation from triplicate measurements. (.1) and (.3) indicate 0.1 and 0.3 mg dosage respectively.

Application of two concentrations (0.1 and 0.3 mg) of crude extracts of terrestrial (FRR5152) and 2009 dust storm originated (ASBS) *A. sydowii* had a minor effect on maximum quantum yield (*F_v_*/*F_m_*) of CS156 *Symbiodinium* (clade C) at day 2 (*F*(4,10) = 27.2, *p* < 0.001), exhibiting a significantly reduced *F_v_*/*F_m_* by 0.025 to 0.056, respectively, compared to the control (Figure 4). *F_v_*/*F_m_* is a measure of the maximum efficiency of photosystem II, used as an index of plant photosynthetic performance [17]. At day 4, 0.1 mg FRR5152 and ASBS crude extracts exhibited significantly higher *F_v_*/*F_m_* of CS156 with increases of 0.051-0.054, whereas 0.3 mg FRR5152 and ASBS crude extracts continued to exhibit significantly lower *F_v_*/*F_m_* at day 4, with declines of 0.029-0.039 compared to control values (*F*(4,10) = 50.2, *p* < 0.001). At days 6 and 8, there was no significant difference in *F_v_*/*F_m_* between treatment and control. However, there was a trend that *F_v_*/*F_m_* at 0.3 mg ASBS crude exhibited lowest values throughout the experiment period. There was no significant effect of crude extracts on the more resilient strain CS163 *Symbiodinium* (clade A1) *F_v_*/*F_m_* throughout the 8-day experimental period (results not shown).

### 2.6. Effect of Known A. Sydowii Metabolites on Symbiodinium Photo-Physiological State

**Figure 5:**
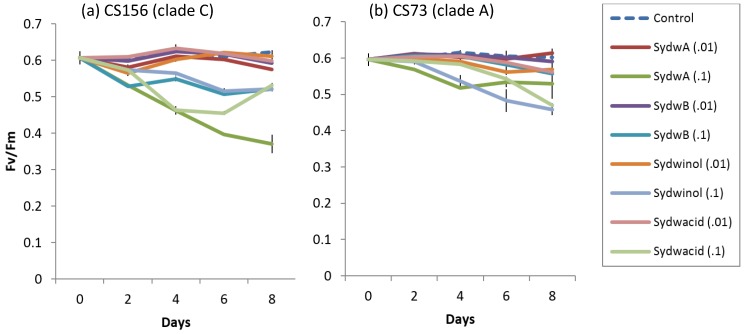
Effect of typical *A. sydowii* metabolites on CS156 (**a**) and CS73 (**b**) *Symbiodinium* maximum fluorescent yield (*F_v_*/*F_m_*) during an eight-day study period. Error bars are sample standard deviation from triplicate measurements. SydwA, SydwB, Sydwinol, and Sydwacid are sydowinin A, sydowinin B, sydowinol and sydowic acid, respectively. (0.01) and (0.1) indicate 0.01 and 0.1 mg dosage applied in this study.

Known *A. sydowii* metabolites had significant effects on *F_v_*/*F_m_* of CS156 (clade C) and CS73 (clade A), however caused no effect on CS163 (clade A1) (Figure 5). Treatment with 0.1 mg doses of typical metabolites significantly reduced *F_v_*/*F_m_* of CS156, whereas 0.01 mg dosage had no or minor effect on *F_v_*/*F_m_* on CS156 (from day 2–8, *F*(8,18) = 65.58 *p* < 0.001, at day 2 *F*(8,18) = 254.6, *p* < 0.001 at day 4, *F*(8,18) = 1060.0, *p* < 0.001 at day 6 and *F*(8,18) = 253.9, *p* < 0.001 at day 8). *F_v_*/*F_m_* of CS156 at 0.1 mg sydowinin A exhibited a gradual decline and lowest *F_v_*/*F_m_* of 0.370 ± 0.0256 at day 8. Application of 0.1 mg of sydowic acid also significantly decreased *F_v_*/*F_m_* to 0.455 ± 0.0061 at day 6. There was an increase at day 8 to 0.532 ± 0.0053. Application of 0.1 mg of sydowinin B caused a significant reduction in *F_v_*/*F_m_* to between 0.507 ± 0.0009 and 0.549 ± 0.0018, and application of sydowinol caused a reduction between 0.516 ± 0.0077 and 0.573 ± 0.016. Application of 0.01 mg of those metabolites caused a minor reduction in *F_v_*/*F_m_* of CS156 by 0.028 to 0.048 compared to the control. On the other hand, 0.1 mg of all typical *A. sydowii* metabolites except sydowinin B caused a significant effect on *F_v_*/*F_m_* of CS73, whereas 0.01 mg of metabolites had no significant effect from day 2 to day 8, *F*(8,18) = 16.8, *p* < 0.001, *F*(8,18) = 45.5, *p* < 0.001, *F*(8,18) = 19.54, *p* < 0.001 and *F*(8,18) = 24.14, *p* < 0.001). At day 2, only 0.1 mg sydowinin A caused a significant decline in *F_v_*/*F_m_* of CS73. After day 2 application of 0.1 mg of sydowinin A, sydowinol and sydowic acid significantly decreased *F_v_*/*F_m_* of CS73 to 0.517 ± 0.0031 (day 4), 0.458 ± 0.0157 (day 8) and 0.470 ± 0.0056 (day 8) respectively.

There was no significant effect from standard metabolites on CS163 *Symbiodinium* *F_v_*/*F_m_* throughout the eight-day experimental period (results not shown), except that at day 4. *F_v_*/*F_m_* of CS163 at 0.1 mg sydowinol caused significantly lower values than those at other treatments with a reduction of 0.041 compared to the control.

## 3. Discussion

We show that the Australian marine *A. sydowii* strains share major metabolites and display comparable metabolic diversity to Australian terrestrial strains and to strains pathogenic to Caribbean coral. We also find secondary colonisation of the rafts by other *Aspergillus* species and fungal strains of *Cladosporium* and *Penicillium*. Our bioassays reveal that the dust-derived marine fungal extracts adversely affect photophysiological performance of the important coral reef dinoflagellate endosymbiont *Symbiodinium*. Different *Symbiodinium* clades exhibit varying sensitivities, mimicking sensitivity to coral bleaching phenomena.

### 3.1. Dust Generated Microbial Raft Ecosystem

Dust has been widely recognised as an important source of nutrient input into the marine environment and a vector for transporting pathogenic microorganisms [18]. The dust storm in 2009 covered a large area of the Australian coast, and created a distinctive marine raft micro-environment.

It is probable that the dust layer was sufficiently hydrophobic to remain un-wetted on the ocean surface for a considerable time, during which spores of many genera of saprophytic species germinated. The levels of fungal spores on dust should reflect typical levels and diversity of soil fungi and have a density of 10^4^ to 10^6^ spores per gram (colony forming units) with considerable species diversity >>100 [19]. Surface culture of fungi is itself not new; forming a raft on liquid media dates to the discovery of penicillin and is a fundamental technique in fungal cultivation. Most, but not all, saprophytic fungi form rafts on stationary liquid culture and these surfaces are profoundly water repellent. In cultivation studies using cellophane rafts on agar media, it was noted that many fungi grown on concentrated media (hypertonic, hyperosmotic, high nutrient) simply did not produce secondary metabolites, however, interceding with a thin cellophane membrane led to massive increases in the abundance and diversity of secondary metabolites [20].

Based on viable spores recovered from trawling silks of the raft masses, *A. sydowii* was the dominant species, with additional species of *Aspergillus*, *Penicillium* and *Cladosporium* accumulated as secondary colonisers. These fungi are well known as terrestrial species, but are also occasionally isolated from marine environments [21,22]. Two previous studies have reported the commonly observed taxa, *Aspergillus* spp., *Cladosporium* spp. and *Penicillium* spp., in the Australian marine environment from sources such as sediment, algae and invertebrates [22,23]. Furthermore, Toledo-Hernández *et al.* isolated *Penicillium* spp. from both healthy and unhealthy coral *Gorgonia ventalina*, which is one of the most abundant species in Puerto Rico [24]. These authors similarly reported that both *Penicillium* and *Aspergillus* were the most abundant fungal species in the corals sampled. Fungal communities are a normal feature of healthy reefs, but the occurrence of massive dust-induced fungal rafts may lead to an infection event.

### 3.2. Secondary Metabolites Associated with Pathogenic and Non-Pathogenic Strains of A. Sydowii

While the HPLC traces of terrestrial and marine *A. sydowii* show some differences in their co-metabolite profiles, the major known metabolites, constituting >50% of the total co-metabolite AUC, are shared. Importantly, the metabolites reflect our general understanding of *A. sydowii* chemistry. Most obvious are the highly characteristic UV spectra of the sydonic acids, sydowinin and sydonols. These metabolites are the chemical framework of every strain investigated in this study and while the strains all share this profile, they are not identical. *A. sydowii* is capable of producing large numbers of secondary metabolites, but most are detectable in only trace levels. Currently, >30 metabolites from *A. sydowii* strains have been published in the literature, however the role and bioactivity of these are largely undescribed. The Australian marine strain displayed a more streamlined metabolite distribution, which suggests intensive strain selection on marine adaptation. A similar trend has been reported by Malmstrøm *et al.* who detected similar co-metabolite profiles between pathogenic and non-pathogenic strains [16].

### 3.3. Effect of Fungal Crude Extracts and A. Sydowii Typical Metabolites on Symbiodinium Photophysiology

*F_v_*/*F_m_* is an indicator of the efficiency of photosystem II charge separation, which determines photophysiological performance of algal species [17]. A decrease in *F_v_*/*F_m_* implies stress to photochemical efficiency [17,25]. Declines in *Symbiodinium F_v_*/*F_m_* might be significant to the coral host, as endosymbiont photosynthesis supports coral metabolism and provides a source for carbon [26]. Our result is consistent with a study that compared *in situ* *F_v_*/*F_m_* of *Symbiodinium* from growth anomaly diseased and healthy coral individuals with a reduction of 1.5 in *F_v_*/*F_m_* [25]. Similarly, other coral disease studies revealed that *Vibrio* bacteria, a pathogen of yellow band disease, caused significantly decreased *Symbiodinium* chlorophyll *a* and *c_2_*, and increased occurrence of lysed cells of *Symbiodinium* [27,28]. In addition, aspergillosis-infected coral tissue harboured fewer *Symbiodinium* cells compared to healthy coral tissues [29]. These findings add support to our conclusion that *Symbiodinium* coral endosymbionts are negatively impacted by typical *A. sydowii* fungal metabolites.

The bioassay results showed that typical *A. sydowii* metabolites, including sydowinin A, sydowinin B, sydowinol and sydowic acid, significantly reduced *F_v_*/*F_m_* of *Symbiodinium* dinoflagellates. Sydowinin A (impacting on CS156 clade C) and sydowinol and sydowic acid (impacting on C73 clade A) were the most active. Previously, *A. sydowii* from the marine sponge *Spongia obscura* has infected the *Gorgonia ventalina*, and this strain produced typical metabolites such as sydonic acid and sydowic acids, 6-*O*-methylsydonic acid, 6-*O*-methyl-13-hydroxysydonic acid and diorcinol [30]. This suggests that pathogenicity could be determined by secondary metabolites. The crude extract bioassays exhibited less clear effects on *Symbiodinium* *F_v_*/*F_m_*, which might be due to the presence of different interacting compounds. However, there was a trend that marine-originated *A. sydowii (*ASBS) had more impact on *Symbiodinium* than the terrestrial strain.

*Symbiodinium* is known to be extremely diverse genetically; eight distinct genetic clades (A–H) have been characterised [31]. This genetic variation plays key functional roles in behavioural, biochemical and physiological variation of coral hosts [32,33]. Our study revealed a difference in responses between *Symbiodinium* clades. Strains CS156 clade C and CS73 clade A exhibited high and moderate sensitivities, respectively, whereas CS163 clade A1 showed low sensitivity. Similarly, *Symbiodinium* stress-tolerant clade A and E dominated the bleached tissues of yellow blotch diseased corals whereas narrowly adapted specialists clade B and C dominated healthy tissues of corals [34]. In contrast, growth anomaly coral disease and sea fan aspergillosis harboured consistent *Symbiodinium* clade types regardless of disease infection status [25,29]. Caribbean octocorals harbour mostly *Symbiodinium* clade B and rarely clade C, whereas those from the Australian Great Barrier Reef (GBR) harbour predominantly clades C and D with clades A, B, G much less abundant [35]. Clade B might be as sensitive as clade C towards typical *A. sydowii* metabolites since they are closely related genetically and have been defined as narrowly adapted specialists causing yellow blotch disease. Furthermore, clade C was the most sensitive *Symbiodinium* clade in this study. The fact that the Great Barrier Reef has not experienced significant fungal coral disease events to date [36] suggest that the high diversity of octocoral communities reflects a less impacted coral reef community compared to the Caribbean.

## 4. Experimental Section

### 4.1. Fungal Isolation from the Continuous Plankton Recorder Silks and A. Sydowii Strains

Fungi were isolated from the formalin-preserved Continuous Plankton Recorder (CPR) silks by scraping spores from the silks and inoculating them on MEA, or producing aqueous spore suspensions as follows. Approximately 5 × 5 cm of the CPR silk samples were added to 2 mL of autoclaved sterile Milli-Q water and vortexed for 20 s to remove embedded spores and mycelium from the plankton silk. The suspension was centrifuged at 10,000× *g* for 5 min. Supernatants were removed and the pellet re-suspended in 2 mL of Milli-Q water. Spore suspensions or spores floating on top of Milli-Q water were inoculated on either full strength, or half strength MEA with tetracycline (40 µg/mL) using a 10-µL disposal-inoculating loop. Morphologically different fungal colonies were selected and their metabolite profiles analyzed by HPLC.

*A. sydowii* strains from non-marine environments were supplied by Dr. John Pitt and Dr. Ailsa Hocking, Commonwealth Scientific and Industrial Research Organisation (CSIRO) Food Research North Ryde (FRR) Culture Collection (Supplementary Material, Table S1). Two *A. sydowii* strains isolated from diseased sea fan corals (FK1) were supplied by Prof. Drew Harvell, Cornell University, USA.

### 4.2. HPLC Analysis on Fungal Secondary Metabolites

Fungal cultures from the CPR silk after the dust storm in 2009 and five strains from CSIRO were grown in a wide range of solid media. The agars, Czapek-dox agar (CZA; 30 g Sucrose, 3 g Sodium nitrate, 1 g Di-potassium phosphate, 0.5 g Magnesium sulphate, 0.5 g Potassium chloride, 0.01 g iron(II) Sulphate heptahydrate, 15 g Agar, 1L distilled water), malt extract agar (MEA; 20 g Malt extract, 20 g Glucose, 1g Peptone, 20 g Agar, 1L distilled water), yeast extract and sucrose agar (YES; 20 g Yeast extract, 150 g Sucrose, 20 g Agar, 1L distilled water), and glycerol casein agar (GCA; 10 g Glycerol, 0.3 g Casein, 0.3 g Potassium nitrate, 2 g Sodium chloride, 2 g Dipotassium phosphate, 0.05 g Magnesum sulfate heptahydrate, 0.02 g Calcium carbonate, 0.01 g Iron(II) sulphate heptahydrate, 18 g agar, 1 L distilled water) were prepared. The grains, barley, rice (jasmine and basmati) and cracked wheat were prepared by hydration (~30 g with 30 mL water in a 250 mL flask) during sterilisation (120 °C for 40 min.). The agar and grains were inoculated with a suspension of fungal spores and incubated at 24 °C and sampled at seven and 14 days. Subsamples (1 g) of the cultures were extracted with methanol (2 mL) for a minimum of 1 h on a wrist shaker, centrifuged (15,700 × *g* for 3 min., Eppendorf) and analysed by HPLC. Small sections of silk material (locations 2 and 3) were also directly extracted with methanol and processed by HPLC.

Analytical HPLC was performed on a gradient Shimadzu HPLC system comprising an LC-10AT VP gradient chromatograph, SPD-M10A VP diode array detector and SCL-10A VP system controller. The column used was an Alltima C_18_ rocket format column (100 Å, 53 × 7 mm, 3 µm; Grace Discovery, Deerfield, IL, USA) eluted with a 3 mL/min gradient of 10%–100% MeCN/H_2_O (+0.01% TFA) over 7 min. The HPLC traces were accessioned into our in-house database, COMET [37] and the major metabolites were analysed by retention time and UV-Vis spectrum fit against a library of known metabolites (>25,000 spectra) and type species library (>25,000 spectra from 2000 fungal species).

HPLC-DAD-ESI(±)MS was performed on an Agilent 1260 UHPLC coupled to an Agilent 6130B single quadrupole mass spectrometer. The column was an Agilent Zorbax Rapid Resolution HT Eclipse Plus C_18_ (50 × 2.1 mm, 1.8 µm) eluted with a 0.5 mL/min gradient of 10%–100% MeCN/H_2_O (+0.025% formic acid) over 10 min.

### 4.3. Symbiodinium Dinoflagellate Strains

Axenic strains of the endosymbiotic dinoflagellate *Symbiodinium* were obtained from the Australian National Algal Culture Collection (ANACC) in Hobart. Strains CS73, CS156 and CS163 were selected based on genetic clades, growth rate and geographic origin (Table 2). Strains were grown in f2 media [38] and maintained at 25 °C under 12/12 h light/dark cycle. Algal cell counts were undertaken using a haemocytometer.

### 4.4. Crude Extracts and Typical A. Sydowii Secondary Metabolites

Four typical *A. sydowii* metabolite standards sydowinol, sydowinin A, sydowinin B and sydowic acid were provided by Professor Hiromitsu Nakajima, Tottori University, Japan. Crystallized metabolites were dissolved in ≥99.9% acetone and diluted to 70% with autoclaved sterile Milli-Q water. Known weights of methanol evaporated crude extracts of day 7 optimisation of FRR 5152 and ASBS were dissolved in 2.6% methanol and sonicated to maximize solubility of metabolites. Crude extracts were then filter-sterilized (Millex GP 0.22 µm).

### 4.5. Symbiodinium Photophysiology Assays

Either 0.01 mg or 0.1 mg of standard *A. sydowii* metabolites (Figure 5), or 0.1 mg or 0.3 mg of FRR5152 or ASBS extracts were added to 24 well microplates (Greiner Bio-one, Frickenhausen, Germany). Solvents were completely evaporated in sterile conditions prior to the addition of 1.0 × 10^5^ cells/mL exponential growth stage *Symbiodinium* dinoflagellate cell culture. The maximum quantum yield (*F_v_*/*F_m_*) of *Symbiodinium* was measured from the bottom of each well using an underwater pulse amplitude modulated fluorometer (Diving-PAM, Walz, Effeltrich, Germany). Algal cultures were dark adapted for 30 to 60 min at 25 °C before each measurement. Instrument gains were adjusted between 1 and 12, and *F*_0_ (background chlorophyll fluorescence) set to the range of 200 to 400. This assay was conducted in triplicate, and average values of three measurements of each replicate were taken. Samples were dark adapted again for second/third measurements. Day 0 indicates before adding the fungal metabolites. This assay was conducted for 8 days, and measurements were taken every second day to follow the impact of fungal metabolites on the dinoflagellate photosynthetic performance.

### 4.6. Statistical Analysis

Statistical analyses were performed using R [39]. One-way analysis of variance (ANOVA) was conducted to test significant differences in maximum quantum yield among treatments each day. When the main effect was significant, Tukey’s honestly significant difference (HSD) *post hoc* tests were conducted. Box–Cox transformation was applied to determine appropriate transformation to improve normality and homogeneity of variance. A significance level of 0.05 was applied.

## 5. Conclusions

The marine strain of *A. sydowii* isolated after a 2009 dust storm on the eastern Australian seaboard represents a streamlined secondary metabolite profile that shares the same major co-metabolites of its terrestrial parent. These major co-metabolites significantly reduced *Symbiodinium* photo-physiological state. This effect may constitute a key mechanism for the effect of sea fan aspergillosis on the coral host. However, there is also evidence that the marine *A. sydowii* is more toxic to *Symbiodinium* than the terrestrial strain, suggesting important roles for other co-metabolites in the *A. sydowii* repertoire. Furthermore, there was a clade-dependent degree of sensitivity of *Symbiodinium* to *A. sydowii* metabolites and crude extract which mimics the sensitivity of corals and their symbionts to coral bleaching. Work in progress aims to characterise novel strain-specific *A. sydowii* metabolites and examine their role in driving differing virulence by terrestrial and marine, Australian and Caribbean fungal strains.

## Figures and Tables

**Table 1:** Fungal strains isolated from 2009 dust storm and secondary metabolites found by HPLC analysis of methanol extracts grown on malt extract agar (MEA). Numbers in brackets indicates the number of each species.

Location	Species	Metabolite(s)	% Isolates
1	*A. sydowii*	sydonic acid	5.3 (2)
		sydonic acid, sydonol	15.8 (6)
		sydonic acid, unknown metabolites^1^	7.9 (3)
	*Penicilllium* sp.	rugulosin	2.6 (1)
	Unknown 1	no detectable metabolites	5.3 (2)
2	*A. sydowii*	sydonic acid	7.9 (3)
		sydonic acid, sydowinin B	2.6 (1)
		sydonic acid, unknown metabolites^1^	10.5 (4)
	*Aspergillus* sp.	sterigmatocystin	2.6 (1)
3	*Penicillium* sp.	rugulosin	2.6 (1)
4	*A. sydowii*	sydonic acid	21.1 (8)
		sydonic acid, sydonol	2.6 (1)
	*Aspergillus* sp.	sterigmatocystin	5.3 (2)
	*Cladosporium* sp.	no detectable metabolites	2.6 (1)
	Unknown 2	unknown metabolite^2^	5.3 (2)
			Total 38 isolates

^1^ λ_max_ 230, 279, 288, 320 and 365 nm; ^2^ λ_max_ 216 nm.

**Table 2:** *Symbiodinium* dinoflagellate strains/clades used in the bioassays.

CS-No.	Clade	Source Location
CS-73	Clade A	Heron Is., Great Barrier Reef, Queensland, Australia
CS-156	Clade C	Hawaii, USA
CS-163	Clade A1	Palau

## Supplementary Materials

The following are available online at http://www.mdpi.com/1660-3397/14/3/59/s1, Table S1: *A. sydowii* isolates examined in this study, Table S2: HPLC/DAD profile of *A. sydowii* FRR5068, Table S3: HPLC/DAD profile of *A. sydowii* ASBS, Table S4: HPLC/DAD profile of *A. sydowii* FK1, Figure S1: HPLC profiles of the terrestrial *A. sydowii* investigated in the present study, Figure S2: HPLC profiles of extracted silks from location 2 and 3.
