# Supplementary material for: Aspergillus Sydowii Marine Fungal Bloom in Australian Coastal Waters, Its Metabolites and Potential Impact on Symbiodinium Dinoflagellates
Source: Mar Drugs. 2016 Mar 16;14(3):59. doi: 10.3390/md14030059 (PMC4810073; doi:10.3390/md14030059)
Supplement: Supplementary file 1 [file marinedrugs-14-00059-s001.pdf]

Supplementary Materials: *Aspergillus Sydowii* Marine Fungal Bloom in Australian Coastal Waters, Its Metabolites and Potential Impact on *Symbiodinium* Dinoflagellates

Aiko Hayashi [1], Andrew Crombie [2], Ernest Lacey [2], Anthony J. Richardson [3,4], Daniel Vuong [2], Andrew M. Piggott [5] and Gustaaf Hallegraeff [1,*]

Table S1. *A. sydowii* isolates examined in this study.

ID	Source	Location
FRR34	Wheat or flour sample	Not available
FRR2972	Dried fish; *Puntius javanicus*	Kalibaru, Indonesia
FRR2991	Dried fish; *Sardinella fimbriata*	Bogor, Indonesia
FRR4822	Partially dried sultana grapes	Mildura, VIC
FRR5068	Air, wardroom duct	Naval vessel
FRR5136	Water bath with ethylene glycol	Maribyrnong, VIC
FRR5152	Water bath with ethylene glycol additive	Maribyrnong, VIC
ASBS[1]	CPR silk after dust storm in 2009	Between Brisbane and Sydney
FK1	Diseased *Gorgonia ventalina*	Key West, Florida

[1] defined as the original *A. sydowii* culture isolated in 2009.

Table S2. HPLC/DAD profile of *A. sydowii* FRR5068.

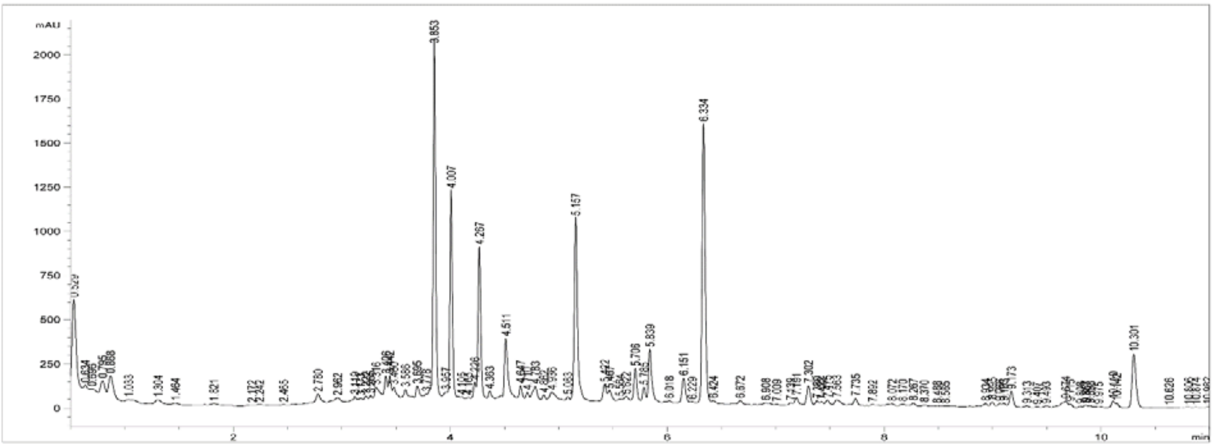

Peak Number	Ret. Time	Area	% Area	Cumulative % Area	Peak Number	Ret. Time	Area	% Area	Cumulative % Area
1	0.53	1,273	6.61	6.61	45	5.62	40	0.21	66.61
2	0.63	34	0.17	6.78	46	5.71	331	1.72	68.33
3	0.70	21	0.11	6.89	47	5.79	99	0.51	68.84
4	0.79	157	0.82	7.71	48	5.84	542	2.81	71.65
5	0.87	290	1.51	9.21	49	6.02	15	0.08	71.73
6	1.03	76	0.39	9.61	50	6.15	306	1.59	73.32
7	1.30	91	0.47	10.08	51	6.23	5	0.03	73.35
8	1.46	31	0.16	10.24	52	6.33	3,078	15.97	89.32
9	1.82	34	0.18	10.42	53	6.42	14	0.07	89.39
10	2.17	7	0.03	10.45	54	6.67	68	0.35	89.75
11	2.24	11	0.06	10.51	55	6.91	8	0.04	89.79
12	2.46	8	0.04	10.55	56	7.01	8	0.04	89.83
13	2.78	161	0.84	11.39	57	7.14	9	0.05	89.88

14	2.96	98	0.51	11.89	58	7.19	64	0.33	90.21
15	3.12	27	0.14	12.04	59	7.30	236	1.22	91.43
16	3.17	8	0.04	12.08	60	7.38	21	0.11	91.54
17	3.22	21	0.11	12.19	61	7.42	10	0.05	91.59
18	3.26	18	0.09	12.28	62	7.48	38	0.20	91.79
19	3.32	131	0.68	12.96	63	7.56	88	0.46	92.25
20	3.41	98	0.51	13.47	64	7.73	73	0.38	92.63
21	3.44	85	0.44	13.90	65	7.89	8	0.04	92.67
22	3.48	36	0.19	14.09	66	8.07	39	0.20	92.87
23	3.59	98	0.51	14.60	67	8.17	16	0.09	92.96
24	3.69	124	0.64	15.24	68	8.27	27	0.14	93.10
25	3.78	6	0.03	15.27	69	8.37	17	0.09	93.19
26	3.85	3,143	16.31	31.58	70	8.49	7	0.04	93.22
27	3.96	12	0.06	31.64	71	8.56	7	0.04	93.26
28	4.01	1,760	9.13	40.77	72	8.93	30	0.16	93.42
29	4.11	6	0.03	40.80	73	9.00	39	0.20	93.62
30	4.17	9	0.05	40.85	74	9.07	9	0.05	93.67
31	4.23	51	0.26	41.11	75	9.10	9	0.05	93.71
32	4.27	1,324	6.87	47.98	76	9.17	170	0.88	94.60
33	4.36	68	0.35	48.34	77	9.31	20	0.11	94.70
34	4.51	701	3.64	51.97	78	9.41	6	0.03	94.74
35	4.65	111	0.58	52.55	79	9.49	19	0.10	94.83
36	4.71	44	0.23	52.78	80	9.67	75	0.39	95.22
37	4.78	172	0.89	53.67	81	9.72	12	0.06	95.28
38	4.86	35	0.18	53.86	82	9.81	7	0.04	95.31
39	4.94	144	0.75	54.60	83	9.86	12	0.06	95.37
40	5.08	21	0.11	54.71	84	9.90	6	0.03	95.40
41	5.16	2,072	10.75	65.47	85	9.97	11	0.06	95.46
42	5.42	119	0.62	66.09	86	10.11	33	0.17	95.63
43	5.47	53	0.27	66.36	87	10.14	9	0.05	95.68
44	5.56	9	0.04	66.40	88	10.30	779	4.04	99.72

Table S3. HPLC/DAD profile of *A. sydowii* ASBS.

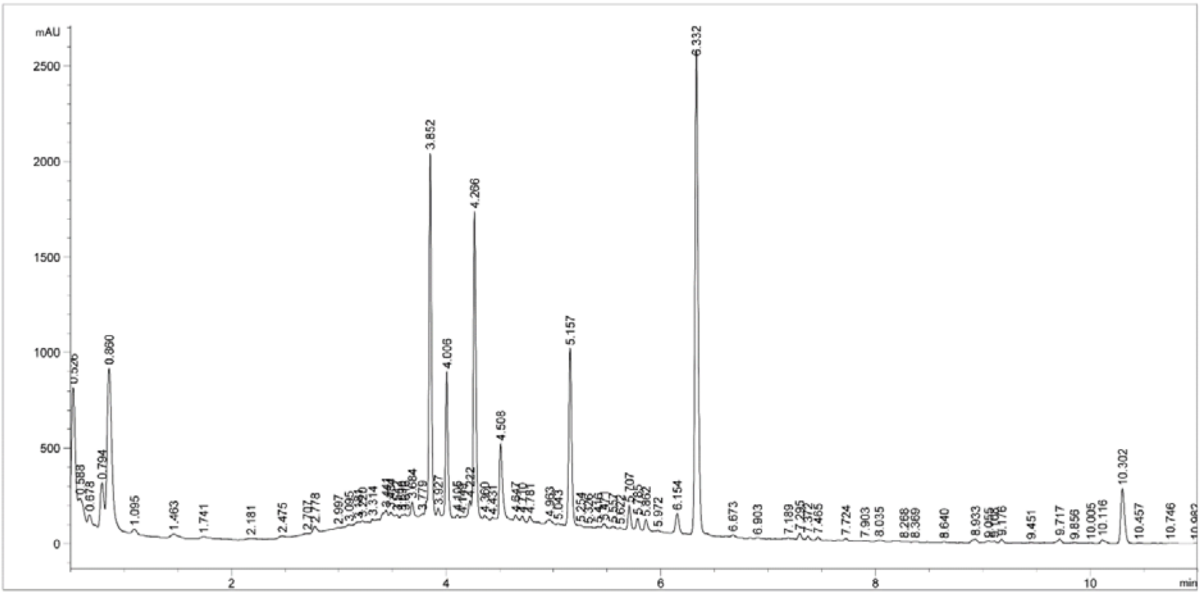

Peak Number	Ret. Time	Area	% Area	Cumulative % Area	Peak Number	Ret. Time	Area	% Area	Cumulative % Area
1	0.53	1,309	6.09	6.09	40	5.16	1,710	7.96	66.25
2	0.59	111	0.51	6.61	41	5.25	6	0.03	66.27
3	0.68	83	0.39	7.00	42	5.33	9	0.04	66.32

4	0.79	343	1.60	8.59	43	5.42	10	0.05	66.36
5	0.86	2,109	9.82	18.41	44	5.47	40	0.19	66.55
6	1.09	65	0.30	18.71	45	5.56	25	0.12	66.67
7	1.46	88	0.41	19.12	46	5.62	9	0.04	66.71
8	1.74	68	0.31	19.44	47	5.71	212	0.99	67.70
9	2.19	32	0.15	19.59	48	5.79	104	0.48	68.18
10	2.48	32	0.15	19.74	49	5.86	114	0.53	68.71
11	2.71	6	0.03	19.76	50	5.97	19	0.09	68.80
12	2.78	79	0.37	20.13	51	6.15	233	1.08	69.88
13	3.00	12	0.06	20.19	52	6.33	5,152	23.98	93.86
14	3.09	17	0.08	20.27	53	6.67	63	0.30	94.16
15	3.18	13	0.06	20.33	54	6.90	11	0.05	94.21
16	3.22	10	0.05	20.38	55	7.19	13	0.06	94.27
17	3.31	14	0.07	20.44	56	7.30	60	0.28	94.55
18	3.44	99	0.46	20.90	57	7.37	33	0.15	94.70
19	3.48	29	0.14	21.04	58	7.46	31	0.14	94.85
20	3.54	17	0.08	21.12	59	7.72	23	0.11	94.95
21	3.59	9	0.04	21.16	60	7.90	9	0.04	94.99
22	3.62	9	0.04	21.20	61	8.03	30	0.14	95.13
23	3.68	129	0.60	21.80	62	8.27	6	0.03	95.16
24	3.78	7	0.03	21.83	63	8.37	7	0.03	95.19
25	3.85	2,966	13.81	35.64	64	8.64	16	0.07	95.27
26	3.93	54	0.25	35.89	65	8.93	62	0.29	95.55
27	4.01	1,177	5.48	41.37	66	9.05	12	0.06	95.61
28	4.11	25	0.12	41.49	67	9.11	8	0.04	95.65
29	4.15	8	0.04	41.53	68	9.18	38	0.18	95.83
30	4.22	50	0.23	41.76	69	9.45	8	0.04	95.86
31	4.27	2,482	11.55	53.31	70	9.72	62	0.29	96.15
32	4.36	34	0.16	53.47	71	9.86	14	0.07	96.22
33	4.43	12	0.05	53.52	72	10.01	11	0.05	96.27
34	4.51	810	3.77	57.29	73	10.12	64	0.30	96.56
35	4.65	39	0.18	57.48	74	10.30	716	3.33	99.90
36	4.71	37	0.17	57.65	75	10.46	7	0.03	99.93
37	4.78	56	0.26	57.91	76	10.75	6	0.03	99.96
38	4.96	73	0.34	58.25	77	10.98	8	0.04	100.00
39	5.04	7	0.03	58.29					

Table S4. HPLC/DAD profile of *A. sydowii* FK1.

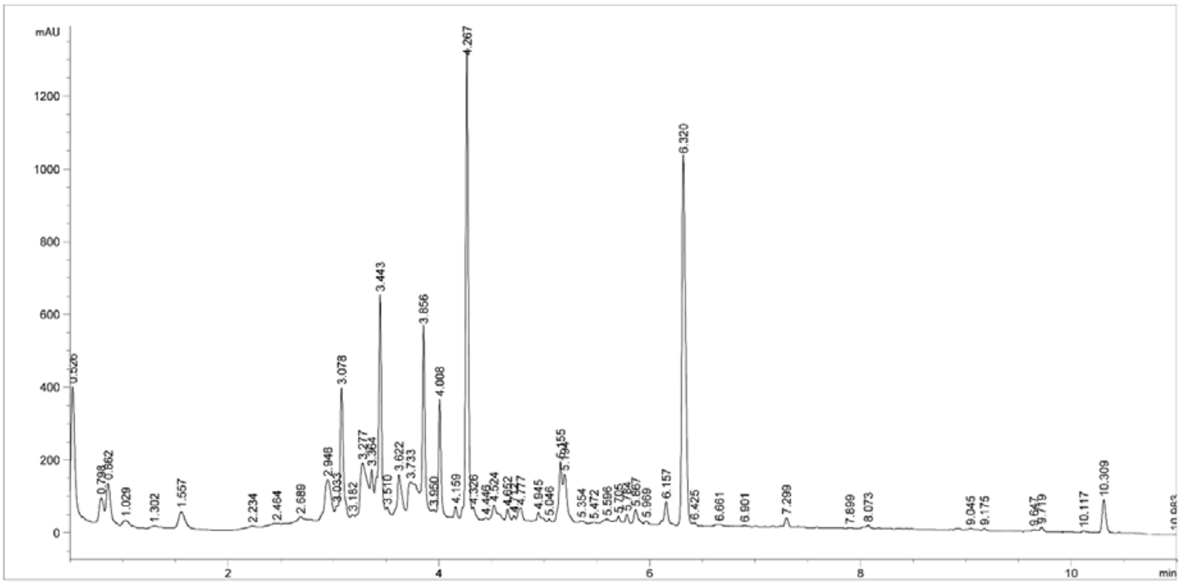

Peak Number	Ret. Time	Area	% Area	Cumulative % Area	Peak Number	Ret. Time	Area	% Area	Cumulative % Area
1	0.53	840	7.25	7.25	29	4.71	11	0.09	70.09
2	0.80	112	0.97	8.22	30	4.78	83	0.71	70.80
3	0.86	200	1.73	9.96	31	4.94	27	0.23	71.03
4	1.03	66	0.57	10.53	32	5.05	9	0.08	71.11
5	1.30	31	0.27	10.80	33	5.15	123	1.06	72.17
6	1.56	212	1.83	12.63	34	5.19	94	0.81	72.98
7	2.23	35	0.31	12.93	35	5.35	15	0.13	73.11
8	2.46	24	0.21	13.14	36	5.47	10	0.09	73.20
9	2.69	46	0.40	13.54	37	5.60	31	0.26	73.47
10	2.95	368	3.17	16.71	38	5.71	20	0.17	73.64
11	3.03	7	0.06	16.77	39	5.78	39	0.34	73.97
12	3.08	655	5.66	22.43	40	5.87	94	0.81	74.78
13	3.18	7	0.06	22.49	41	5.97	14	0.12	74.90
14	3.28	371	3.20	25.70	42	6.16	107	0.92	75.82
15	3.36	71	0.61	26.31	43	6.32	2,405	20.76	96.58
16	3.44	986	8.51	34.82	44	6.43	9	0.07	96.66
17	3.51	12	0.11	34.92	45	6.62	9	0.08	96.73
18	3.62	272	2.35	37.27	46	6.90	10	0.09	96.82
19	3.73	271	2.34	39.61	47	7.30	54	0.46	97.28
20	3.86	707	6.11	45.72	48	7.90	8	0.07	97.36
21	3.95	7	0.06	45.77	49	8.07	13	0.11	97.47
22	4.01	479	4.13	49.91	50	9.05	18	0.16	97.62
23	4.16	51	0.44	50.35	51	9.18	9	0.07	97.70
24	4.27	2,149	18.56	68.91	52	9.65	8	0.07	97.76
25	4.33	13	0.12	69.03	53	9.72	22	0.19	97.95
26	4.45	6	0.05	69.07	54	10.12	14	0.12	98.08
27	4.52	62	0.53	69.61	55	10.31	217	1.87	99.95
28	4.65	45	0.39	70.00	56	10.98	6	0.05	100.00

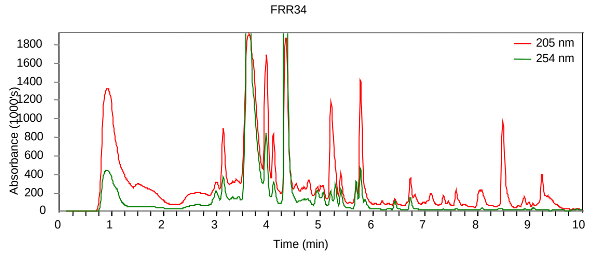

(**A**)

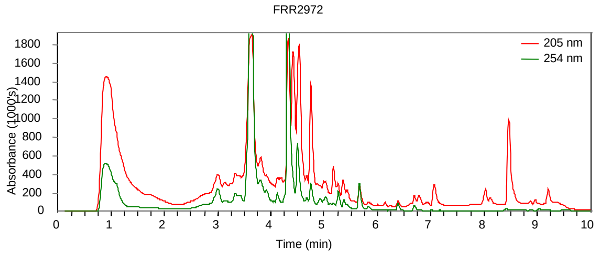

(**B**)

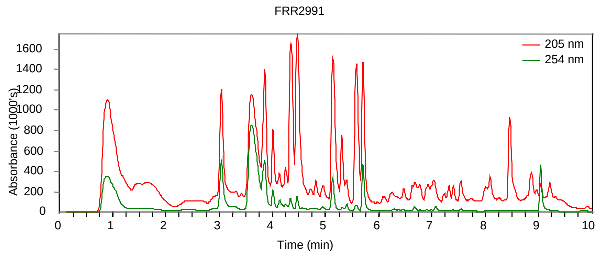

(**C**)

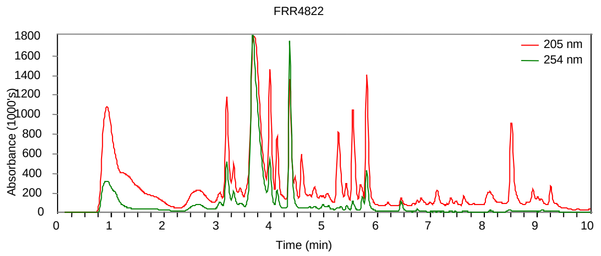

(**D**)

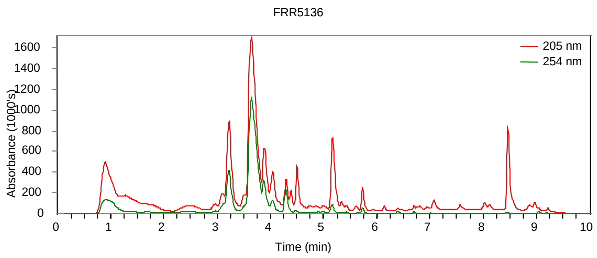

(**E**)

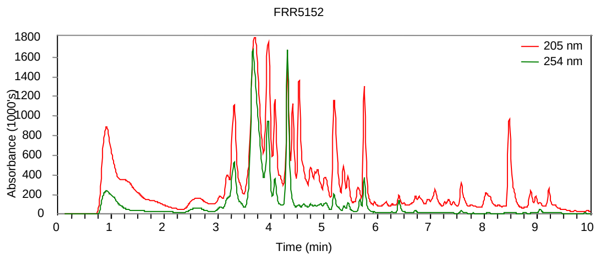

(**F**)

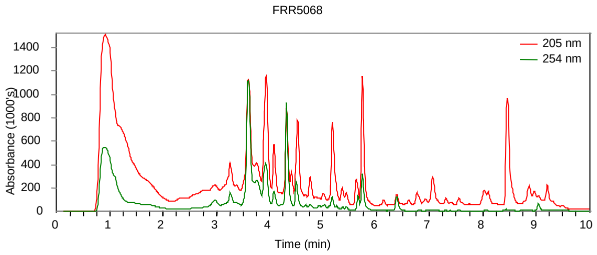

(**G**)

Figure S1. HPLC profiles of *A. sydowii* investigated in the present study. The strains were cultured on rice for 7 days extracted with methanol (1 gm/mL) and analysed by gradient HPLC, (conditioned as described in body of the article). A. FRR34; B. FRR2972; C. FRR2991; D. FRR4822; E. FRR5136; F. FRR5152 and G. FRR5068.

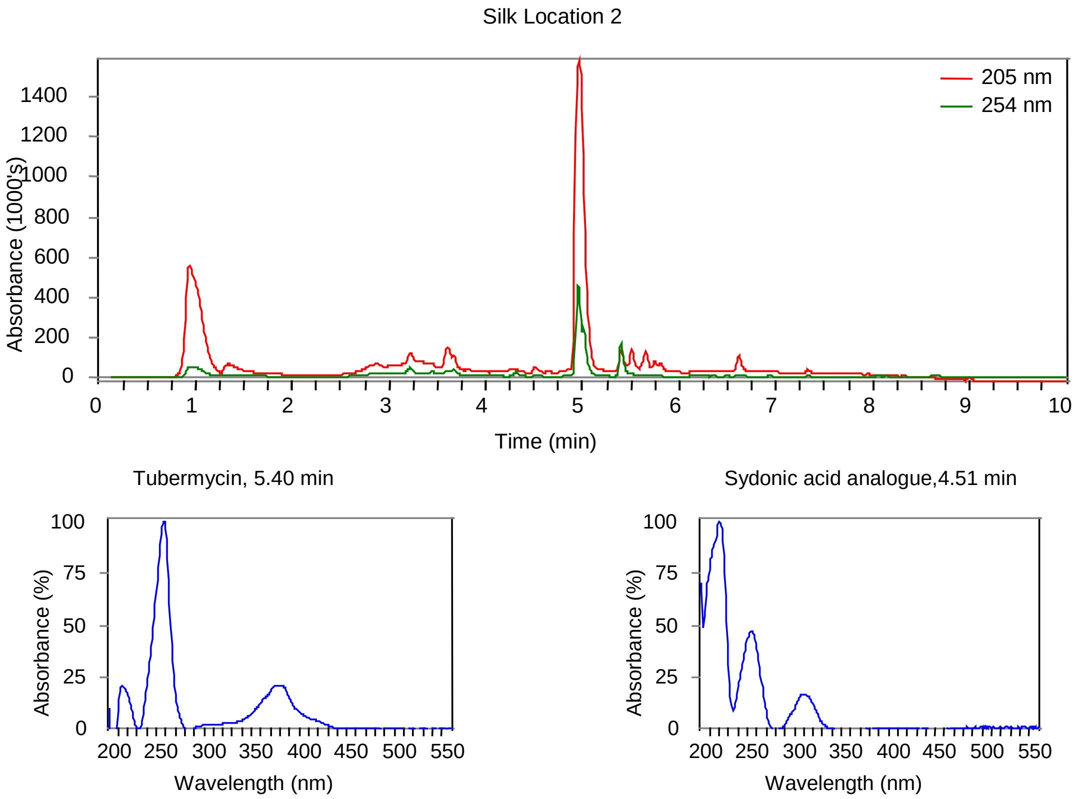

(**A**)

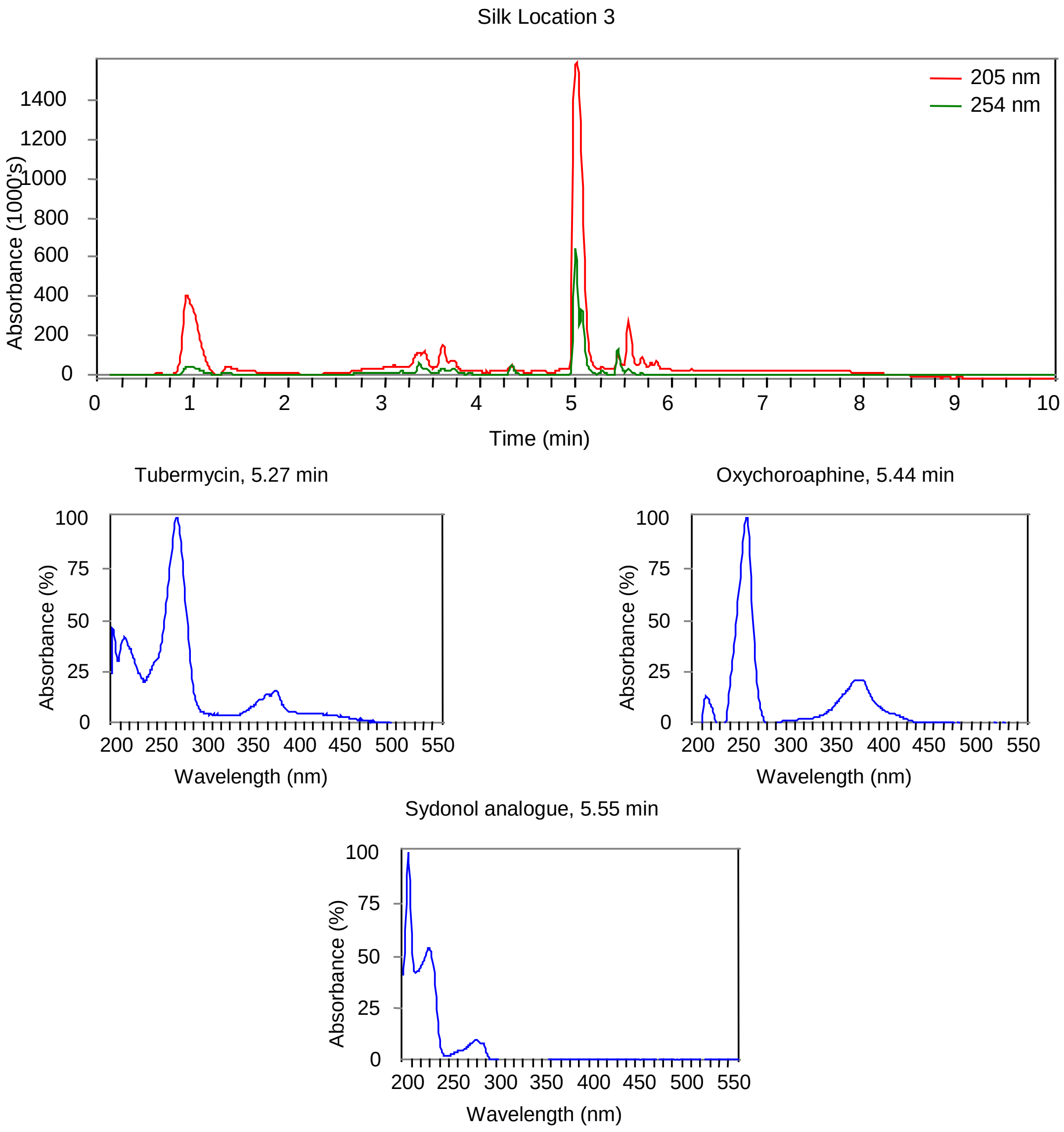

(**B**)

Figure S2. HPLC profiles of extracted silks from (**A**). location 2 and (**B**). location 3, with the UV spectra of the microbial metabolites in the extract. (Note: The dominant peak eluting at 5 min, is an artefact of extraction of the silks with methanol).
